# Revisiting the fourth dimension of tool use: how objects become tools for capuchin monkeys

**DOI:** 10.1017/ehs.2021.16

**Published:** 2021-03-05

**Authors:** Briseida Resende, Andrès Ballesteros-Ardilla, Dorothy Fragaszy, Elisabetta Visalberghi, Patrícia Izar

**Affiliations:** 1 Instituto de Psicologia, Universidade de São Paulo, SP, Brasil; 2 Psychology Department, University of Georgia, Athens, GA, USA; 3 Institute of Cognitive Sciences and Technologies, National Research Council, Rome, Italy

**Keywords:** animal traditions, practice, development, tool, artefact

## Abstract

Culture allows humans to adapt to a diversity of contexts. Participatory experience in technical activities and activity with artefacts provide the basis for learning traditional technical skills. Some populations of non-human animals use tools. The ways in which artefacts influence the development of a traditional skill in non-human species can provide insight into essential supports for technical traditions in humans and shared learning processes across species. In wild bearded capuchins, nut cracking leaves edible pieces of nuts, nut shells and stones used as hammers at anvil sites. We addressed how mastery of cracking nuts by young monkeys is associated with interactions with these objects. We studied monkeys’ reuse of nuts, hammers and anvils and the outcome of attempts to crack nuts, and from these data derived their behavioural variability and proficiency in nut cracking. Behavioural variability was the most robust predictor of whether a monkey collects pieces of nuts cracked by others or reuses stones and nuts, and was a stronger predictor of proficiency than age. Young monkeys were increasingly likely to reuse the stone used by another after the other monkey had left the anvil as they increasingly focused their behaviour on actions relevant to cracking nuts.

## Graphical abstract

**Figure:**
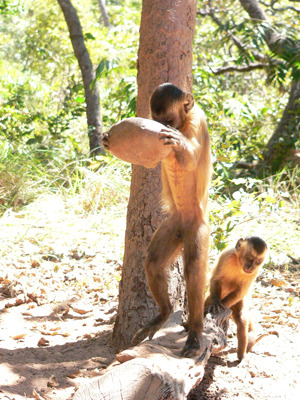

**Social media summary:** The more focused on nutcracking activities wild capuchins are, the more they reuse their social mates’ tools.

## Introduction

Culture allows humans to adapt to an extreme diversity of habitats and contexts. It involves the horizontal and vertical social transmission of technological improvements, facilitating the development of sophisticated tools (Legare, 2017). Learning technical skills is an essential outcome of human culture. Children grow up in environments modified by human activity, with objects that make up local traditions. Furniture, shelters, cutlery, clothes and tools of various types are ubiquitous. Children participate while adults use tools, eventually learning to use them on their own.

### Artefacts and traditions

In many cultures, parents actively organise space to guide children's learning to use objects in culturally appropriate ways, providing them with the opportunity to learn how to use objects even in the absence of a demonstration (Flynn et al., 2013). Even when adults do not actively organise space for children's learning, social context influences what children learn about objects. For example, using eye tracker and goal-directed gaze, Green et al. (2016) compared the skills of Swedish and Chinese infants in predicting how adults manipulate spoons and chopsticks. Chinese infants moved their gaze to the adult's mouth when chopsticks were used, but not spoons, while Swedish infants did the same when a spoon was used, but not chopsticks. Thus, infants’ gaze was affected by their experience with adults using these objects according to their own tradition even before they began to handle them themselves: the cultural context provided children with different artefacts and ways to deal with them.

This report concerns the relations among the actions of young bearded capuchin monkeys (*Sapajus libidinosus*) with objects associated with nut cracking across the several years that they are developing this skill. Nut cracking is a technical tradition, and as in many human technical traditions, it results in enduring artefacts – the stones and anvils used as hammers (i.e. the tools). According to Borgo et al. (2013), tools are ‘physical artefacts whose attributed capacity is related to the purpose of physically acting on some other entity’. We use the term ‘artefact’ *sensu* Borgo et al. (2013) for the stones and anvils used in nut cracking. Nut cracking by capuchins, platyrrhine monkeys distantly related to apes and humans, offers a comparative reference point for consideration of the role of artefacts and partners in learning a technical skill.

Understanding how non-human animals acquire technical traditions may provide insights into the evolution of human cultural learning (Musgrave et al., 2020a). Fragaszy et al. (2013), using niche construction theory (Fragaszy, 2011; Odling-Smee et al., 2003), proposed that enduring artefacts support non-human primates’ learning technical skills. In such cases, there is an important temporal dimension in learning, because the durability of the artefact allows the learner to use the object used previously as a tool by another. For example, infant chimpanzees (*Pan troglodytes*) reuse folded leaf that others have used to collect water (Sousa et al., 2009), reuse plant probes used by others to collect termites (Musgrave et al., 2020b) and reuse stones as hammers for cracking nuts (Estienne et al., 2019). Thus, skilled adults affect infant chimpanzees’ learning of how objects can be used as tools by attracting attention to these objects and by providing access to them for individual activity, as described for human infants (Rakoczy et al., 2005). Japanese macaques (*Macaca fuscata*) learning to handle stones (Leca et al., 2010) provide another example. Some groups of these monkeys have traditions of handling stones in particular ways (Huffman & Hirata, 2003). Young monkeys are attracted to accumulations of stones at specific locations that remain from adults’ handling sessions. The presence of durable artefacts at specific locations supports the reuse of stones over time and the maintenance of the stone-handling tradition in groups of Japanese macaques.

According to Ingold (2001), skills are achieved through a process of enskilment. Ingold states that skills do not simply appear. Rather, capacities and forms of organisms emerge from developmental processes, from the sensorial engagement of the organism in a highly structured environment. Skills are continually generated and regenerated in the developmental contexts of each organism: neuromuscular systems and motor habits are formed while the competencies are established through the organisms’ actions in their environment. In this view, development is the result of active engagement in dynamic activities (Thelen et al., 1991). The activities and practices of one generation shape the environment for the next, giving the next generation an education of attention (Ingold, 2001). Each generation builds environmental contexts through their embodied activities, where the next generation will develop its own embodied skills. This conception of development applies to humans and non-human animals alike. Technical traditions in non-human animals, such as capuchins cracking nuts with stone tools, are examples of this process.

### Nut cracking in capuchin monkeys

Several wild populations of robust capuchin monkeys (genus *Sapajus*) crack open nuts or other encased food using hard objects, such as stones or wood, as hammers and anvils (review in Izar et al., 2018). Young monkeys engage in actions with nuts, anvils and hammers over several years before they can open nuts (Eshchar et al., 2016; Resende et al., 2008). Monkeys must follow a specific action sequence to crack a nut (take the nut, place it on the anvil, take the hammer and hit it on the nut). In Resende et al.'s (2014) study of a free-ranging group of capuchin monkeys, young monkeys initially performed these actions in variable order and also performed other, unrelated actions such as rolling a nut (Resende et al., 2014). As they became more skilled at cracking nuts, the monkeys performed the relevant actions in a consistent and effective order (place the nut on a hard surface, pick up a stone, strike the nut with the stone) and performed irrelevant actions less often (i.e. behavioural variability declined). Behavioural variability was a key predictor of the monkeys’ proficiency at cracking nuts.

Beyond adopting a consistent sequence of actions with nuts and stones, to crack a nut, capuchins must control the movement of their body and of the hammer (as one system) so as to produce strikes with sufficient kinetic energy at impact to break the shell. This aspect of nut cracking requires both strength and skill (Mangalam et al., 2016, 2018, 2020). Skill at cracking nuts is not in the monkeys’ nervous system, nor in the environment, but in their integration. As monkeys act with nuts and stones, they produce different types of space and force relations among stones, nuts, surfaces and their bodies. Through cycles of perception and action, they learn these relations’ affordances, and how to coordinate them, as humans do (Thelen et al., 1991; Ulrich and Wolff, 1991; Lockman, 2000).

Nut cracking, like all foraging activities in capuchin monkeys, takes place in a social setting. Ottoni et al. (2005) reported that, during cracking bouts, young monkeys preferentially watched skillful conspecifics cracking and profited from scrounging the remains of nuts at the anvil site. Coelho et al. (2015) confirmed that young monkeys chose which monkeys to observe, and found that young monkeys more frequently observed more productive crackers that also tolerated the young monkeys scrounging. This suggests that eating pieces of cracked nuts attracts young monkeys to particular crackers while they are cracking.

At a longer time scale, Eshchar et al. (2016) showed that at one site, Fazenda Boa Vista, enduring artefacts (hammer and anvil stones) at the nut-cracking sites, as well as enduring cracked shells, allowed unskilled group members to act with these objects in the absence of an individual cracking nuts. The environment formed by the activity of skilled group members biases the attention of unskilled members and directs their manipulative activities towards the elements linked to cracking nuts. As stones suitable for cracking the resistant palm nuts are rare in the landscape at Fazenda Boa Vista (Visalberghi et al., 2009), and they are too heavy for young monkeys to transport them, the presence of stones at anvil sites constitutes an important modification of the environment aiding young monkeys’ handling them together with nuts. Eshchar et al. (2016; see also Fragaszy et al., 2017) demonstrated that young capuchin monkeys were attracted to the cracking artefacts and manipulated nuts and hammer stones at above-baseline rates when others were cracking nuts. After others ceased cracking, young capuchins continued these actions at rates higher than during baseline for periods ranging from 30 seconds to several minutes, depending on the action, suggesting that others’ actions facilitated their actions. They also increased actions with nuts when at or within arm's reach of anvil sites, suggesting that the artefacts alone stimulate activities related to cracking. All of these studies have addressed the role of group members’ actions and their artefacts on attracting the attention and action of non-skilled immatures. However, developmental changes related to the reuse of artefacts and to the consumption of leftovers have not been reported.

The study reported here extends Resende et al.'s (2014) study relating behavioural variability and the emergence of skilled use of a tool in a wild population of bearded capuchin monkeys (*S. libidinosus*), and addresses hypotheses relating to reuse of nuts and stones by monkeys learning to crack nuts. Specifically, we carried out a longitudinal study examining the relative proportion of all visits to anvil sites in which young monkeys scrounged (finding edible pieces of nut kernels; hereafter, leftovers) and reused artefacts. We registered if the reuse was immediate, while the previous cracker was still at the site, or within 30 seconds after that monkey left the site. We also registered if monkeys collected pieces of cracked nuts immediately after the nut was cracked and whether this occurred in the presence of the cracker (immediate scrounging) or after the other monkey had left (delayed scrounging). We use the term ‘scrounge’ without implying generosity from the individual that cracked the nut, nor that the action occurred at the expense of that individual. We interpreted activities towards nuts and scrounging as revealing perception of food, and activities towards anvils and hammers as revealing perception of tools. We included individuals’ age (in months), which is a correlate of body size (Fragaszy et al*.,* 2016), variability of behaviours in nut cracking, and proficiency at cracking as independent variables in our analyses.

Considering the previously reported inverse relation between proficiency in nut cracking and variability of actions related to nut cracking (Resende et al., 2014), we predicted that: (a) proportions of visits to anvil sites where the young monkey displayed scrounging and reuse of nuts (abandoned or partially cracked by the previous monkey) are positively correlated with behavioural variability during cracking activity and negatively correlated with age and proficiency; (b) proportions of visits to anvil sites where the young monkey displayed immediate reuse of hammer and anvil are positively associated with behavioural variability and negatively associated with age and proficiency; and (c) proportions of visits to anvil sites where the young monkey displayed delayed reuse of hammer and anvil show the opposite pattern – negative association with behavioural variability and positive association with age and proficiency.

## Methods

### Study site

This study was conducted at Fazenda Boa Vista (hereafter, FBV; 9°39′ S, 45°25′ W), in the state of Piauí, Brazil. FBV is a flat open woodland (altitude 420 m a.s.l.). Rainfall is concentrated mainly between October and April (for further information see Eshchar et al., 2016; Visalberghi et al., 2008). Palm trees are abundant in FBV, and in this study monkeys cracked piassava nuts (*Orbygnia* sp.; average length 61.3 mm, 50.6 g; the shell is 6 mm thick with a peak-force-at-failure of 11.5 kN; Visalberghi et al., 2008).

The hammer stones used as tools to crack nuts weigh on average around 1 kg, although they range from 250 g to 2.5 kg. They are quartzites, siltstones or hard sandstones. Anvils are flat, or nearly flat, horizontal or have slightly sloping surfaces – a boulder, an exposed stone or a horizontal log (for details, see Visalberghi et al., 2007).

**Figure 1.**
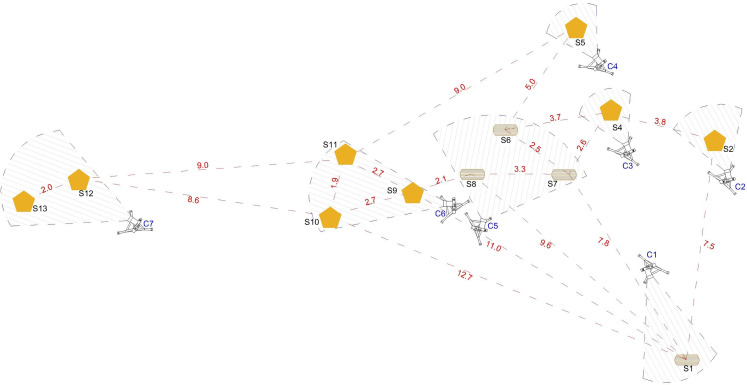
This picture shows the 12 anvils in the outdoor laboratory area where the study was conducted, the locations of the seven cameras and the view that each camera captured. S, Site; C, camera; pentagons, stone anvils; cylinders, wooden anvils; red dotted lines, distance (m) between anvils; blue dotted lines, shooting angle of each camera.

The study took place in the field laboratory, an open area about 12 m in diameter that capuchin monkeys regularly and freely visited (more details in Fragaszy et al., 2013). The field laboratory contained 12 cracking sites (four wooden and eight stone anvils) with stones nearby that were commonly used by proficient individuals for cracking nuts (Visalberghi & Fragaszy, 2013) and nut shells all over the area (Figure 1).

### Subjects and data collection

The 33 subjects providing data in this study ranged in age from 3 months to more than 10 years (for those adult monkeys for which we did not know the exact age, we assigned an age of 120 months for data analysis). Seven monkeys were born and three left the group during the study (see Supplementary Material).

Data were collected in four discrete field seasons (dry season – season 1, June–July 2016 and season 3, August–September 2017; rainy season – season 2, February–March 2017 and season 4, April–May 2018), each lasting 34, 33, 31 and 20 days respectively. In season 1, there were 23 monkeys (seven adult females, five adult males, three immature males and eight immature females); in season 2 there were 23 monkeys (seven adult females, four adult males, four immature males and eight immature females); in season 3, there were 28 monkeys (seven adult females, four adult males, eight immature males and nine immature females) and in season 4 there were 30 monkeys (seven adult females, four adult males, nine immature males and 10 immature females). Because the participation of the monkeys was opportunistic, the number of sessions and minutes of engagement in cracking activity varied across seasons (season 1, 12 sessions and 592 min; season 2, 11 sessions and 420 min; season 3, five sessions and 219 min; season 4, 12 sessions and 614 min).

**Figure 2.**
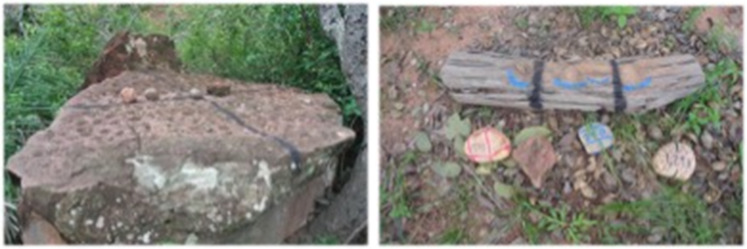
Stone anvil (left) and wooden anvil (right) with the stone kits (light, 300–800 g; medium, 800–1400 g; heavy, >1400 g; and sandstone, inadequately hard for cracking). The black marks on anvils delimit the subareas. The blue marks on the anvils show the pits left by repeated cracking on the same spot. Similar pits are evident on the surface of the stone anvil.

We used 12 cracking sites (four wooden and eight stone anvils; Figure 1). Each anvil was identified and subdivided into small areas with black ink marks to facilitate recording of reuse (Figure 2): we registered as anvil reuse when the focal animal used the same subarea as the previous monkey. We marked stones used as hammers. We ran one or two experimental sessions per day, depending on the monkeys’ attendance at the field site. Prior to each experimental session, we put a hammer kit with four stones differing in size and material at each cracking site (Figure 2). Three researchers turned the cameras on and set the experimental setting. Before starting each new session, the researchers set out the hammer kit and 30 nuts on the field laboratory area. We used seven cameras strategically positioned to capture all of the cracking activity going on at the field laboratory (Figure 1). The session started when the monkeys approached the area. While recording, the researchers spoke the names of the monkeys that arrived at the field laboratory and the anvils they used, for later reference during video transcription. Filming ended when all of the nuts had been cracked and/or when all of the monkeys left the field laboratory, whichever came first. At the completion of the study, all marks were removed from the anvils and hammer stones.

The protocol was reviewed and approved by the CEUA IPUSP (Ethical Committee for Use of Animals from Instituto de Psicologia da USP, CEUA 6870180216). This research was conducted under ICMBio permit no. 28689-5. The study adheres to the code of best practices for studies of non-human primates set by the International Primatological Society.

### Data transcription and variables

Details about the subjects’ participation are provided in Supplementary Material. The videos were transcribed using BORIS software, a free and open-source software available for GNU/Linux, Windows and Mac OS (http://www.boris.unito.it/). For each monkey who appeared in the video, we registered if it was following a group member and if it manipulated hammers, anvils and nuts. If so, we registered how they manipulated nuts, anvils and hammers, according to the following categories: take, tap, rub, bite, lick, sniff, rotate, hit (using the hands, the nut or the hammer), place nut on the anvil. We registered if each nut was cracked open, and if the manipulated item was the same as the one used by the immediately previous cracker who used the site in that session. We registered the reuse of nuts when the focal monkeys acted on nuts that were abandoned intact or partially cracked by the cracker. We registered the cracker's identity, and if the reuse was immediate (while the cracker was still on the anvil) or delayed (up to 30 seconds after the cracker had left the site). We also registered if the monkey scrounged (i.e. ingested nut leftovers) while the monkey that had just cracked a nut was still at the site (immediate scrounging), or up to 30 seconds after it had left (delayed scrounging), if the young monkey had observed the cracker at close range (positioning the body facing the cracker, gazing at its action), and if there were agonistic interactions. Since there were few agonistic events, they were not included in further analysis.

We defined the reuse index (RI) and the scrounging index (SI) as: RI = frequency of visits with reuse/frequency of visits and SI = frequency of visits with scrounging/frequency of visits. These indexes were calculated for each monkey, in each season. The closer to 1 they were, the more the monkey reused/scrounged in every visit to the sites. We calculated indexes for ‘immediate reuse’, when the cracker was present, and ‘delayed reuse’, up to 30 seconds after the cracker had left the cracking site.

We calculated, for each monkey, in each season in which it appeared during data collection, the RI values for immediate and delayed hammer reuse, immediate and delayed anvil reuse and immediate and delayed nut reuse (abandoned intact or partially cracked), as well as the total RI (hammer and anvil reuse). Also, we calculated, for each monkey, in each season in which it appeared during data collection, the SI for: immediate and delayed scrounging and the total SI. Thus we had eight values for SI (4 seasons × 2 types of scrounging – immediate and delayed) for each animal that appeared at an anvil in all four seasons.

For testing behavioural variability considering actions filmed at the cracking sites, we calculated the behavioural variability index (BVI) for each monkey in each season (Resende et al., 2014), as:
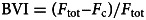
where *F*_c_ is the frequency of nut-cracking behaviour and *F*_tot_ is the frequency of manipulative behaviour.

A BVI near 1 means a higher proportion of different categories of behaviour performed at the sites, indicating that the monkeys’ actions were not restricted to cracking activity; a BVI near 0 means that the animal restricted its actions to the sequence used for cracking.

For testing proficiency, we calculated, for each monkey in each season, a proficiency index (PI) (Fragaszy et al., 2010):
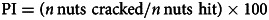


In order to distinguish individuals that never struck a nut with a stone from those that struck a nut with a stone but did not crack it, we added the constant 0.0001 to the number of cracked nuts of those that struck but never cracked. Thus, 0 means that the monkey never struck a nut with a stone.

### Analyses

The data comprised 59 seasonal samples for immatures (3–72 months) and 39 seasonal samples for adults (73 months and older). We analysed the proportion of monkeys’ visits to cracking sites per season in which they scrounged edible leftovers or reused artefacts of conspecifics’ cracking activities. Independent variables were level of cracking skills, age, behavioural variability and the presence of the previous cracker (present during immediate reuse; absent during delayed reuse). We tested three predictions: (a) the proportion of visits to anvil sites in which the monkeys displayed scrounging and reuse of nuts are positively correlated with behavioural variability during cracking activity and negatively correlated with age and proficiency; (b) the proportion of visits to anvil sites in which the monkeys displayed immediate reuse of hammer and anvil are positively associated with behavioural variability and negatively associated with age and proficiency; and (c) the proportion of visits to anvil sites in which the monkeys displayed delayed reuse of hammer and anvil show the opposite pattern – negative association with behavioural variability and positive association with age and proficiency.

Our analyses used the monkeys’ ages (in months), BVI and PI as developmental categories indicating cracking skills (regressors) in generalised linear mixed models, an extension of generalised linear models. We assumed a normal distribution with a log link to regress the independent variables age, BVI and PI on the dependent variables: immediate reuse of hammer stones, immediate reuse of anvil stones, immediate reuse of nuts and immediate scrounging, and delayed reuse of hammer stones, delayed reuse of anvil stones, delayed reuse of nuts and delayed scrounging. Because the individuals in our sample were unevenly sampled across the four field seasons (some disappeared from the study group and others were born during the study), we controlled for individual identity and field season. We tested each independent variable alone and in combination and we chose the best models based on Akaike information criterion values. Statistical tests were conducted using IBM SPSS Statistics for Windows, version 21.0.

## Results

**Figure 3.**
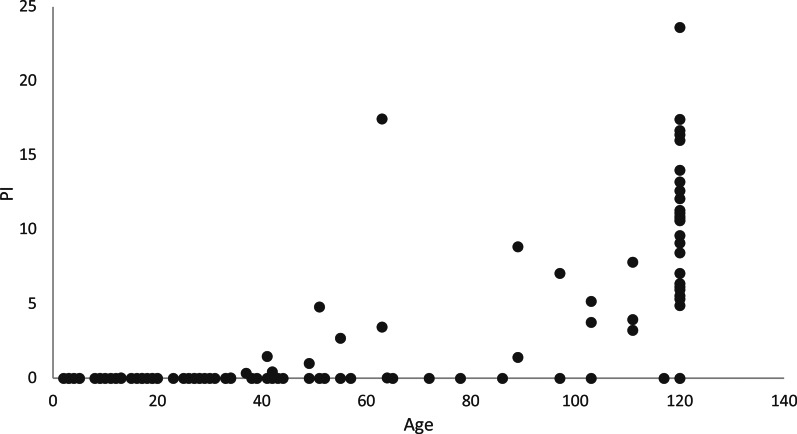
Distribution of proficiency index (PI) in nut cracking as a function of age (in months). Each point represents one monkey during one season (98 samples in total). Adults were assigned age = 120 months.

**Figure 4.**
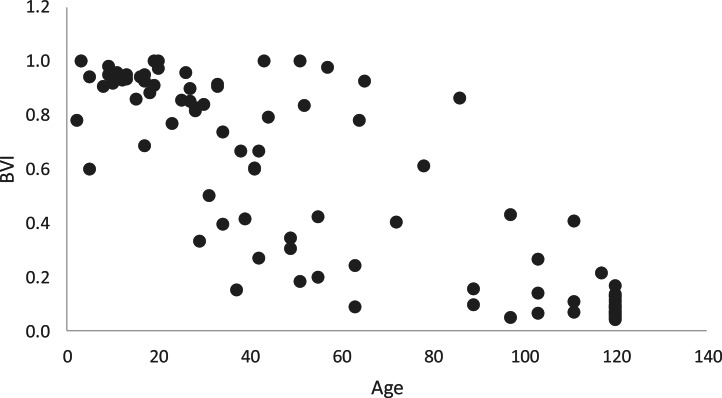
Distribution of behavioural variability index (BVI) in nut cracking as a function of age (in months). Each point represents one monkey during one season (98 samples in total). Adults were assigned age = 120 months.

The distributions of PI and BVI as a function of age are shown in Figures 3 and 4. Adult monkeys had a PI greater than 0 in 85% of samples (average PI = 7.7); average BVI was 0.15. Immatures had PI greater than 0 in just 20% of samples and average BVI was 0.73. Age predicted PI (*F* = 114.74, d.f._1_ = 1, d.f._2_ = 98, *p* < 0.001), although it was only slightly positively related (coefficient = 0.082; SD = 0.008, *t* = 10.71, *p* < 0.001). Similarly, age was a significant predictor of BVI (*F* = 99.89, d.f._1_ = 1, d.f._2_ = 98, *p* < 0.001), but it was only slightly negatively related (coefficient = −0.015; SD = 0.002, *t* = −9.99, *p* < 0.001). The BVI, on the other hand, was a significant predictor of PI (*F* = 27.86, d.f._1_ = 1, d.f._2_ = 98, *p* < 0.001) and was strongly negatively related to PI (coefficient = −3.30, SD = 0.63, *t* = −5.28, *p* < 0.001). Thus, BVI was a better predictor of proficiency than age.

### Scrounging and reuse of nuts

**Figure 5.**
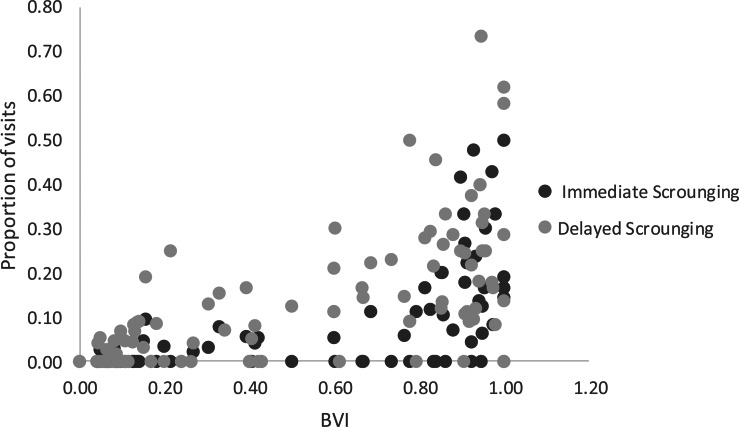
Distribution of proportion of visits with immediate and delayed scrounging in relation to the BVI. Each point represents one monkey during one season (98 samples in total).

Adults were observed engaging in immediate scrounging at least once in three samples (7%), and in delayed scrounging in 17 samples (41%). Immatures engaged in immediate scrounging in 78% of samples, and delayed scrounging in 86% of samples. The age of individuals and their BVI were significant regressors of immediate scrounging (*F* = 14.27; d.f._1_ = 2; d.f._2_ = 97, *p* < 0.001). The BVI contributed most to the regression and was positively related (coefficient = 2.91, SD = 0.82, *t* = 3.56, *p* = 0.001, 95% CI = 1.287–4.537; see Figure 5), while age was slightly negatively related to immediate scrounging (coefficient = −0.015, SD = 0.005, *t* = −2.84, *p* = 0.006, 95% CI = −0.028 to −0.004). The BVI was the best regressor of delayed scrounging (*F* = 25.27, d.f._1_ = 1, d.f._2_ = 98, *p* < 0.001) and was positively related to this variable (coefficient = 2.03, SD = 0.40, *t* = 5.03, *p* < 0.001, 95% CI = 1.229–2.832).

**Figure 6.**
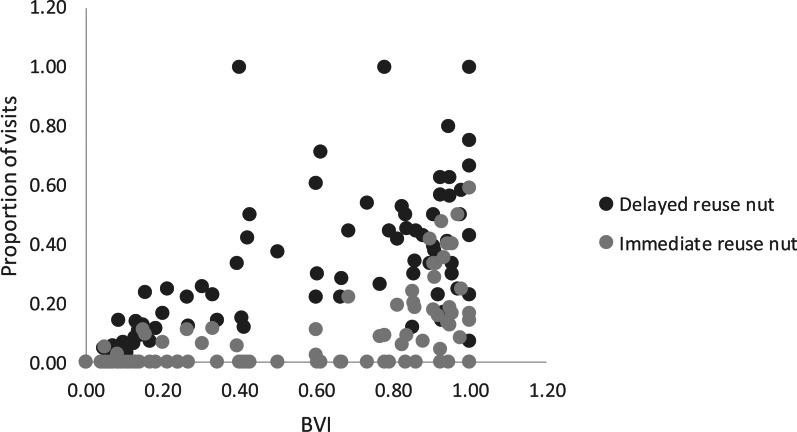
Distribution of proportion of visits with immediate and delayed reuse of nuts in relation to the BVI. Each point represents one monkey during one season (98 samples in total).

Adults were observed at least once reusing nuts immediately in two samples (5%) and immatures in 61% of samples. The ages of individuals and their BVIs were significant regressors of immediate reuse of nuts (*F* = 12.69; d.f._1_ = 2; d.f._2_ = 97, *p* < 0.001). The BVI contributed most to the regression and was positively related (coefficient = 2.53, SD = 0.81, *t* = 3.13, *p* = 0.002, 95% CI = 0.928–4.138), while age was slightly negatively related to immediate reuse of nuts (coefficient = −0.016, SD = 0.006, *t* = −2.76, *p* = 0.007, 95% CI = −0.028 to −0.004). Adults were observed at least once reusing nuts after a delay in 64% of samples and immatures, in 97% of samples. For the delayed reuse of nuts, the best model included the variables BVI and PI as regressors (*F* = 13.24, d.f._1_ = 2, d.f._2_ = 97; *p* < 0.001). The BVI contributed most to the regression and was positively related to the reuse of nuts (coefficient = 0.82, SD = 0.23, *t* = 3.53, *p* = 0.001, 95% CI = 0.357–1.278), while the PI was slightly negatively related to the reuse of nuts (coefficient = −0.22, SD = 0.10, *t* = −2.28, *p* < 0.025, 95% CI = −0.415 to −0.029). In sum, BVI was the strongest predictor of scrounging and of the reuse of nuts (Figure 6).

### Immediate reuse of hammer and anvil

**Figure 7.**
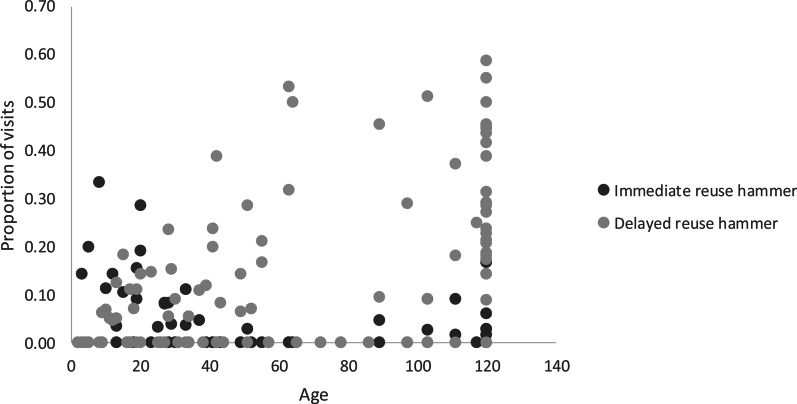
Distribution of proportion of visits with immediate and delayed reuse of hammer stones in relation to age (in months). Each point represents one monkey during one season (98 samples in total). Adults were assigned age = 120 months.

Adults reused hammer stones immediately at least once per season in a total of seven samples (17%); immatures did so in 22 samples (37%). The best model for immediate reuse of hammer stones included only the regressor age of the individuals (*F* = 5.495; d.f._1_ = 1; d.f._2_ = 98, *p* = 0.021). As individuals grew older, they decreased the immediate reuse of hammer stones (coefficient = 0.032, SD = 0.014; *t* = −2.344, *p* = 0.021, 95% CI = −0.059 to −0.005; see Figure 7). Variation in immediate reuse of anvils was not related to any of the regressors tested.

### Delayed reuse of hammers and anvils

Adults reused a stone after a delay at least once per season in 95% of samples; immatures did so in 61% of samples (Figure 7). Adults reused a stone after delay in 26% of visits to an anvil; immatures did so in 9% of visits. All variables were significantly related to the delayed reuse of hammer stone, but when regressed in a single model (*F* = 10.87, d.f._1_ = 3; d.f._2_ = 96, *p* < 0.001), the BVI was the only significant regressor and its coefficient was negative (coefficient = −2.83, SD = 0.65, *t* = −4.68, *p* < 0.001, 95% CI = −0.345 to −0.130). The best model for delayed reuse of anvils included BVI and PI as regressors (*F* = 15.38, d.f._1_ = 2, d.f._2_ = 96; *p* < 0.001), but only the BVI contributed significantly to the regression and was negatively related to the reuse of an anvil (coefficient = −1.57, SD = 0.36, *t* = −4.37, *p* < 0.001, 95% CI = −2.278 to −0.854). In sum, monkeys with high behavioural variability (younger, less proficient monkeys) were unlikely to reuse a stone or an anvil used by another monkey within the previous 30 seconds.

## Discussion

Our study addressed how young capuchin monkeys’ interactions with objects that are associated with the consumption of nuts change over time, as they begin to crack nuts on their own using stone hammers and anvils. We predicted that (a) the proportions of visits to anvil sites in which the young monkey displayed scrounging and reuse of nuts would be positively associated with behavioural variability during cracking activity and negatively associated with age and proficiency, (b) the proportions of visits to anvil sites where the young monkey displayed immediate reuse of hammer and of anvil would be positively associated with behavioural variability and negatively associated with age and proficiency and (c) the proportions of visits to anvil sites where the young monkey displayed delayed reuse of hammer and of anvil would be negatively associated with behavioural variability and positively associated with age and proficiency.

We confirmed the first prediction, and only partially confirmed the second and third. As the monkeys grew older, becoming proficient at cracking nuts and, especially, as they behaved more consistently with stones and nuts, they scrounged nuts less often. Age was the only predictor of immediate reuse of hammer stones – younger monkeys were more likely to do this than older monkeys – but the effect of age was modest. Delayed reuse of hammer stones was predicted only by behavioural variability. The less variable their behaviour towards nuts and stones was, the more likely monkeys were to reuse hammers and anvils after the previous cracker had left the anvil. Neither age nor proficiency predicted delayed reuse of hammer stones.

Overall, young unskilled monkeys show consistent scrounging and reuse of nuts, both immediate and delayed, but re-using stones is not as prominent an activity as looking for nut debris to eat. This finding was expected because very young monkeys are too small to lift most of the hammer stones used by adults. However, as monkeys developed cracking skills (and grew larger), they shifted to the adult pattern of bringing nuts to the anvil and re-using a stone after the previous cracker had left the anvil. Adults reused stones within 30 seconds of a previous cracker leaving the anvil site on about a quarter of their trips to an anvil. Immatures approached this proportion around four years of age. We suggest that monkeys began to value stones as potential tools when they could use them to crack nuts, and in that same time frame, nuts left by others became less valued than nuts that they brought to the anvil themselves.

Perhaps the most robust finding in this study is the predictive relation in young monkeys between decreasing variability in behaviour and developing skill at cracking nuts. The pattern we observed in capuchin monkeys of decreasing behavioural variation with skill development is seen in humans mastering motor tasks, such as Indian craftsmen who use hammering movements for knapping stones (Biryukova & Bril, 2008) or children who show more adaptive movements through practice (Adolph & Berger, 2011), as well as captive adult capuchins learning to crack nuts with tools (Visalberghi, 1987). Tool use emerges from previous existing manual behaviours in humans, as Kahrs et al. (2013) have shown, for example, for the emergence of hammering from banging movements in 6- to 15-month-old infants. According to Lockman and Kahrs (2017, p. 330), ‘there is a developmental synergy between affordance detection and motor learning: as immatures continually explore affordances entailed by object-surface combinations in real time, they tune the actions that will be incorporated into tool use over developmental time. … Collectively, the new research suggests that basic types of tool use in young humans and animals are rooted not in higher order cognitive abilities but shared principles of sensorimotor learning’. The present study contributes to the understanding of the sensorimotor foundations of tooling. Gradual changes in the organisation of actions, accompanied by gradual changes in motor capabilities associated with physical growth, underlie improving skills in young monkeys learning to crack nuts with stone hammers. As adults, these movements reflect motor synergies involving the whole body, and fine control of bipedal balance during vigorous dynamic movements with proportionally heavy stones (Mangalam et al., 2016, 2018, 2020).

Returning to the notion of artefacts, identifying how non-human primates develop relations to artefacts may help to illuminate the social learning processes implicated in traditions and culture. Chimpanzees and capuchin monkeys offer slightly different alternatives. Young chimpanzees are attracted by others using tools and, when they can, they eat pieces of foods remaining at anvil sites when adults, usually their mothers, crack nuts (Inoue-Nakamura and Matsuzawa, 1997). When they are very young, they play with objects that adults used as tools, and preferentially reuse adults’ tools (e.g. stone hammers; Estienne et al., 2019; Carvalho et al., 2009; plant probes; Musgrave et al., 2020b) when they are first learning to use tools. Eventually, however, their reliance on others’ tools diminishes. Before they master cracking nuts, immature capuchin monkeys at FBV display the same general pattern as chimpanzees: attraction to others that are cracking nuts, interest in nut debris and active manipulation of the objects associated with cracking nuts. A major difference, however, is that young capuchin monkeys from FBV cannot at first lift the stones used by adults because they are too heavy. They increasingly reuse stones left at anvils as they grow large enough to do so. Adults of both species also reuse stones used by others, in part because hammer stones are rare away from anvils and because they are costly to transport. A second major difference is that, in chimpanzees, immatures rely on their mothers’ nut cracking for access to relevant objects and pieces of food, whereas immature capuchins rely on other adult individuals, in addition to their mother (Estienne et al., 2019; Fragaszy et al., unpublished data). The facilitative roles of social partners for chimpanzees and for capuchins learning to crack nuts include providing appropriate artefacts in one place coupled with attraction to that place in the form of food (pieces of nuts), and promoting repetition of relevant actions that incorporate artefacts (striking nuts with a stone). These are relatively simple aids for both species, but collectively, they are powerful supports for the the development of this technical tradition, because they bias the learner's attention and activities (Fragaszy et al., 2013, 2017).

## Data Availability

Data have been uploaded to Research Gate Repository, on the link: https://www.researchgate.net/publication/344519878_Resende_Ballesteros-Ardila_Fragaszy_Visalberghi_Izar_Oct_7

## Supplementary material

For supplementary material accompanying this paper visit https://doi.org/10.1017/ehs.2021.16. click here to view supplementary material
