# Supplementary material for: Revisiting the fourth dimension of tool use: how objects become tools for capuchin monkeys
Source: Evol Hum Sci. 2021 Mar 5;3:e18. doi: 10.1017/ehs.2021.16 (PMC10427319; doi:10.1017/ehs.2021.16)
Supplement: Supplementary file 1 [file S2513843X21000165sup001.docx]

**Supplementary Material**

Table 1.

Subjects from the study. We list their identity, sex, date of birth, and the season they participated, with their age in months.

| **ID** | **Sex** | **Date of Birth** | **Season 1**  **(age in months)** | **Season 2**  **(age in months)** | **Season 3**  **(age in months)** | **Season 4**  **(age in months)** |
| --- | --- | --- | --- | --- | --- | --- |
| Acerola | Female | March 2017 | - | - | (5) | (13) |
| Arizo | Male | October 2014 | (20) | (28) | (34) | (42) |
| Cacau | Male | March 2015 | (15) | (23) | (29) | (37) |
| Cachaça | Male | March 2012 | (51) | - | - | - |
| Caititu | Male | June 2017 | - | - | (2) | (10) |
| Cenoura | Female | December 2013 | (30) | (38) | (44) | (52) |
| Chani | Female | February 2011 | (64) | (72) | (78) | (86) |
| Chuchu | Female |  | (120) | (120) | (120) | (120) |
| Dançarina | Female | February 2016 | (4) | (12) | (18) | (26) |
| Dita | Female |  | (120) | (120) | (120) | (120) |
| Divina | Female | November 2012 | (43) | (51) | (57) | (65) |
| Donzela | Female | January 2013 | (41) | (49) | (55) | - |
| Doree | Female | November 2007 | (103) | (111) | (117) | - |
| Dourado | Male | March 2017 | - | - | (5) | (13) |
| Duca | Female | October 2014 | (20) | (28) | (34) | (42) |
| Hortelã | Male | November 2016 | - | (3) | (9) | (17) |
| Jatoba | Male |  | - | (120) | (120) | (120) |
| Kascudo | Male |  | (120) | (120) | (120) | (120) |
| Leonardo | Male |  | (120) | - | - | - |
| Marino | Male | November 2014 | (19) | (27) | (33) | - |
| Menta | Female |  | - | - | - | (3) |
| Michele | Female | December 2016 | - | - | (8) | (16) |
| Oliveira | Female | November 2016 | - | - | (9) | (17) |
| Olivia | Female | January 2015 | (17) | (25) | (31) | (39) |
| Paçoca | Female | January 2009 | (89) | (97) | (103) | (111) |
| Pamonha | Female | January 2009 | (89) | (97) | (103) | (111) |
| Patricia | Female | January 2013 | (41) | (49) | (55) | (63) |
| Peteca | Female | November 2014 | (19) | (27) | (33) | - |
| Piassava | Female |  | (120) | (120) | (120) | (120) |
| Pimenta | Female | May 2017 | - | - | (3) | (11) |
| Presente | Male | March 2011 | (63) | - | - | - |
| Teimoso | Male |  | (120) | (120) | (120) | - |
| Xerife | Male |  | (120) | (120) | (120) | (120) |
